# Economic Conditions at the Time of Birth and Cognitive Abilities Late in Life: Evidence from Ten European Countries

**DOI:** 10.1371/journal.pone.0074915

**Published:** 2013-09-11

**Authors:** Gabriele Doblhammer, Gerard J. van den Berg, Thomas Fritze

**Affiliations:** 1 Institute for Sociology and Demography, University of Rostock, Rostock, Germany; 2 Department of Population Studies, German Center for Neurodegenerative Diseases (DZNE), Bonn & Rostock, Germany; 3 Max Planck Institute for Demographic Research, Rostock, Germany; 4 Rostock Center for the Study of Demographic Change, Rostock, Germany; 5 Department of Economics, University of Mannheim, Mannheim, Germany; 6 Institute for Labor Market Policy Evaluation (IFAU), Uppsala, Sweden; 7 VU University Amsterdam, Amsterdam, The Netherlands; 8 Institute for the Study of Labor (IZA), Bonn, Germany; Chiba University Center for Forensic Mental Health, Japan

## Abstract

With ageing populations, it becomes increasingly important to understand the determinants of cognitive ability among the elderly. We apply survey data of 17,070 respondents from ten countries to examine several domains of cognitive functioning at ages 60+, and we link them to the macro-economic deviations in the year of birth. We find that economic conditions at birth significantly influence cognitive functioning late in life in various domains. Recessions negatively influence numeracy, verbal fluency, recall abilities, as well as the score on the omnibus cognitive indicator. The results are robust; controlling for current characteristics does not change effect sizes and significance. We discuss possible causal social and biological pathways.

## Introduction

Most countries face a shift in the age composition of the population towards higher ages. At the same time elderly individuals experience historically low mortality rates combined with a reduction in the prevalence of disability [1]. In an ageing society elderly individuals are more and more often expected to make their own decisions, which may be impaired by poor cognitive abilities [2]–[4].

Knowledge about the determinants of cognitive status among the elderly facilitates the identification of groups who are particularly at risk. This is also important from a health care policy point of view. After all, the costs of care for cognitively impaired individuals are high [5] and are expected to increase in the upcoming decades.

We examine the role of economic conditions early in life on cognitive functioning at old ages. Severe economic recessions have immediate negative effects on health [6], [7] and may also have negative long-term repercussions. The literature on the developmental origins of diseases provides evidence that exposure to adverse stimuli during the first stages of life may hinder the development of vital organs and the immune system, with irreversible negative effects on health at high ages (see the literature overview in the next section).

Economic conditions in the parents' household at birth and outcomes later in life are jointly dependent on unobserved confounders. We deal with this by using the state of the business cycle early in life as an indicator of economic conditions early in life. This follows [8]–[10], who focus on the effects of conditions at birth on mortality rates later in life. The underlying idea is that birth in a recession causes adverse economic conditions in many households. This may in turn lead to a low quality and/or quantity of nutrition, to adverse housing conditions, and to an enhanced stress level in the household. Birth in a boom year is expected to have the opposite effects. Plausibly, the business cycle does not affect late-life health outcomes in other ways than through its effect on health and abilities around birth. An effect of the business cycle on late-life health outcomes is then evidence of a causal effect of early-life conditions on late-life health. [8] and [10] find significant causal effects on mortality and on cardiovascular mortality, respectively. Similar methodological approaches are used by [11], who demonstrates that survival at ages older than 50 is significantly affected by the season of birth, and by [12] and [13], who use variation in food prices early in life. These studies have in common that they exploit modest fluctuations in early-life conditions, and therefore the results are not driven by extreme events like severe famines or epidemics.

The current elderly were born in times where exposure to a recession was a more intrusive event than nowadays. Generous social safety nets were largely absent. Macro-economic recession and boom periods thus provide a unique opportunity to study the effect of changes in the early-life economic environment on late-life cognition. In many European countries, about three to four economic recession and boom periods can be identified between 1900 and 1945. These include the Great Depression in the early 1930s. However, the timing of boom and recession periods and the general economic development differ between countries, which makes a cross-country study design particularly powerful.

We use data from the Survey of Health, Aging and Retirement in Europe (SHARE) among elderly individuals. This survey is designed to be homogeneous across countries. We use 17,070 respondents from ten countries. We examine several domains of cognitive functioning at ages 60+ and link them to the macro-economic deviations in the year of birth.

The outline of the paper is as follows. First we discuss mechanisms and explanations for the long-run effects on cognitive ability. We also summarize the empirical evidence so far, which in fact mostly concerns outcomes below age 60. Then we present the individual data, the macro-economic indicators, and the empirical strategy of our research. After presenting and discussing the results we provide conclusions.

### Background

Since the seminal studies of [14] and [15] about long-term effects of nutrition and infectious disease early in life on late-life health and morbidity, an extensive literature has been documenting how the environment early in life influences adult health and socioeconomic outcomes.

An important pathway may exist through risk factors of cardiovascular disease later in life which increase the subsequent risk of poor cognitive functioning and dementia [16]. Effects of fetal undernutrition [14] on metabolic adaptation in utero may affect the phenotype such that the risk of cardiovascular disease later in life is increased [17]–[19].

Childhood exposure to disease may trigger a similar pathway. Early infectious exposure can lead to a chronic activation of inflammatory pathways which influence morbidity and mortality in adulthood [20], [21] by increasing the risk for cardiovascular disease, type 2 diabetes and the metabolic syndrome [22]. Childhood exposure to measles and typhoid affect cardiac and respiratory functioning later in life [23], while the exposure to small pox epidemics in the first year of life increases mortality from respiratory diseases at old age [12].

More direct pathways may act through brain development. During infancy and childhood the brain requires a large flow of energy of about half of resting metabolism [24], which may be compromised by nutritional and infectious disease stress [25]. Early childhood may represent a particularly vulnerable time period, as the brain is undergoing rapid neurodevelopmental changes [26]: environmental conditions during the brain development early in life may affect cognitive development and cognitive functioning later in life. For example, [27] show that improved early-life nutrition during the first two years of life has a positive impact on cognitive function in adulthood, even after accounting for the effect of education.

Early-life infections can compromise brain development among children, with some infections resulting in permanent impairment (e.g. the effect of malaria on the developing brain [28]). They can also influence cognitive decline through the effects of inflammation on neurodegenerative disease such as dementia, Alzheimer's disease or Parkinson (see [29], [30] and references therein).

We now zoom into a small set of studies that explicitly relate cognitive functioning later in life with exogenous changes in nutrition and the environment in utero or in the first years of life. In fact, most of the outcomes in these studies are measured for prime-aged adults aged up to 60, which is not the sub-population of primary interest if one aims to study (as we do) mild cognitive impairments among individuals aged 60+. [31] find no effects of exposure to the Dutch Hunger Winter famine during pregnancy on cognitive abilities at ages just below 60, while [32] find an effect on a selective attention task but not on a few other measures. The contextual infant mortality rate and the death rates from typhoid, malaria, measles, influenza, and diarrhea are negatively correlated with cognitive functioning measured as delayed word recall in the Health and Retirement Study [33].

[34] find that among individuals born in the Netherlands under adverse economic conditions as captured by mild exogenous shocks, the decline in mental fitness after experiencing a negative life event at high ages, such as stroke, surgery, illness or death of a family member, is worse. That study focuses on cognitive decline rather than the level, and it uses the Mini Mental State Exam score as main outcome variable, which is more indicative of rather severe mental limitations than of common cognitive impairments. [35] experimentally study effects of mild psychological stress shortly after birth on cognitive outcomes at high ages among rats. They find that mild stress causes declines in memory functioning at high ages and they detect accompanying neurological changes.

A different branch of literature provides evidence for the presence of short-run effects of economic conditions in childhood years on the development of children's cognitive skills (see [36] and the overview in [37]). Such short-run effects may be magnified by their influence on the realized individual level of education, making the effect persistent over time [38]. By analogy to this, the conditions at birth could trigger an indirect pathway in which educational achievement plays a crucial role. There are additional pathways that go from parental socioeconomic status to childhood health and human capital and further on to worse health at high ages, but we do not address these directly in our paper. Our results should still reflect them. Birth in a recession is like an experiment in which parental income is reduced while stress around the time of childbirth is increased [39]–[41].

Another pathway may act through the effect of the business cycle in terms of impaired attachment between the young child and the parent resulting in mental health problems and differences in stress coping strategies (for a review see [42]).

The literature discussed leads us to our hypothesis that boom periods experienced around the time of birth have a positive impact on cognitive abilities late in life while the opposite is true for recessionary periods. For every period in-between conception (or even earlier) to the first years of life there is evidence of long-run effects of the environment on later life health. Given the nature of our data, we are not able to single out particular critical periods. Our paper makes a significant contribution to the literature discussed above, in that we focus on individuals aged 60+ while at the same time we allow for a wide geographical and temporal range of idiosyncratic shocks in early-life conditions. Moreover, we consider mild cognitive impairment outcomes, which are of particular societal relevance because of the fraction of individuals affected.

When testing our hypothesis we control for health characteristics of the respondents. Cardiovascular disease, in particular stroke, hypercholesteremia, diabetes, high blood pressure and obesity have been identified as important risk factors of dementia [43]. This is also true for Parkinson's disease [44] and depression [45]. Mild cognitive impairment and early dementia stages usually do not lead to limitations in the activities of daily living (ADL), severe impairment in cognitive functioning, however, is correlated with an increased number of comorbidities [46] and ADL-limitations [47]. We also control for family status and the number of children to account for the effect of social networks which have shown to influence cognitive function at old age [48], [49].

## Materials and Methods

### Ethics Statement

During waves 1 to 4, SHARE has been repeatedly reviewed and approved by the Ethics Committee of the University of Mannheim and most recently in 2010. In addition wave 4 was reviewed and approved by the Ethics Committee of the Max Planck Society in 2012. All information in SHARE is pseudo-anonymised and therefore the identification of individual persons is not possible. All respondents have been informed about the storage and use of the data and about their right to withdraw their consent. Written consent was given by the respondents for their information to be stored in the database and used for research when required by national or regional data protection laws.

### Data

To measure cognitive functioning at age 60+ we use data from the Survey of Health, Ageing and Retirement in Europe (SHARE). This dataset is designed to follow nationally representative samples of individuals above age 50 over time. The first wave of SHARE was conducted in 2004 and 2005 in Israel and eleven European countries representing Northern, Central and Southern Europe. In total, 31,115 persons were interviewed. The second wave, with 34,415 interviews, was conducted in most countries in 2006 and 2007. SHARELIFE, a retrospective survey was conducted in 2008 and 2009 but does not contain information on cognitive functioning. Between 2010 and 2012 another 59,599 interviews were held within the fourth wave. Three different groups of sampling designs were used. In Denmark and Sweden the sampling was carried out by a stratified simple random sampling from national population registers. In Germany, Italy, Spain, and the Netherlands multi-stage sampling using regional and local population registers were conducted. A single or multi-stage sampling using telephone directories followed by screening in the field was performed in Austria, Belgium and Switzerland. There are only minor differences in the sampling design between the three waves used in this study. The final unit of selection was chosen dependent on the availability of frame data. In Germany, Italy, The Netherlands, Spain and Sweden the individual is the unit of selection, in Austria, Denmark, and Switzerland the household is the final unit. In the first wave all age-eligible persons per sampled household and their partners were selected for an interview. Since the second wave only one age-eligible person per household plus his or her partner have been selected [50]–[52].

In a comparison with cross-national surveys the response rates of the SHARE wave 1 countries were shown to be slightly lower than the rates of the European Community Household Panel (ECHP) and the European Labour Force Survey (EU-LFS) conducted by Eurostat. The rates are substantially higher compared to the response rates of five cross-national scientific surveys: European Social Survey (ESS; at two times), European Value Study (EVS), European Election Study (EES) and International Social Survey Project (ISSP) [53].

In the baseline sampling process of the first wave Switzerland (38.8%) and Belgium (39.2%) have the lowest household response rates, while France (81.0%) and Germany (63.4%) have the highest rates. The high within-household individual response rates reveal a high level of willingness to participate. Between 73.7% (Spain) and 93.3% (Germany) of the individuals have been interviewed. The countries in our study have refreshment samples in the second and fourth waves. Compared to the response rates of the first wave the household response rates in the fourth wave are lower in some countries like France, Denmark or the Netherlands, higher in countries like Switzerland or Spain, or similar like in Belgium. The individual response rates within the households are broadly similar to the first wave.

We use the first, second (Releases 2.5.0) and fourth (Release 1) waves of SHARE and include all countries that participated in all the three waves (i.e. Sweden, Denmark, Austria, Germany, the Netherlands, France, Switzerland, Belgium, Spain, Italy). This enables us to differentiate between age and cohort effects. We only use respondents who participated in the first wave of SHARE or responded for the first time to the second or fourth wave, or were part of the refreshment sample of the second or fourth wave. This design prevents effects of repeated interviewing with respondents knowing the questions and their answers beforehand. [54] shows that the average score of cognitive functioning improves between the first and the second wave which may be the result of panel attrition as well as of repeated interviewing. We exclude cohorts born during wars, since GDP data for war years do not always accurately reflect economic conditions. Altogether, this study comprises 17,070 respondents aged 60+ born in the years 1900–1945 excluding the periods of WWI and WWII for warfaring countries as well as those of the Spanish civil war (Table 1).

**Table 1:** Distribution of respondents with information about their cognitive status by country and wave of SHARE excluding war years; boom and recession periods in the ten SHARE countries; excluded war years.

Country	Distribution of respondents					Boom and recession periods		Excluded war years
	Total	Percent	Wave 1	Wave 2	Wave 4	Boom	Recession	
Austria	1,512	8.86	693	21	798	1912-13; 1927-30; 1939-44	1915-21; 1933-35; 1945-46	1914-1918; 1939-45
Belgium	2,054	12.03	1,481	61	512	1911-13; 1923-24; 1926-30; 1937; 1939	1917-21; 1932; 1941-46	1914-1918; 1940-45
Denmark	1,044	6.12	655	386	3	1911; 1913-14; 19231929-31; 1935-39	1917-22; 1925; 1940-43; 1945	1940-45
France	2,041	11.96	1,041	196	804	1912-13; 1924-26; 1928-30; 1936-39	1910; 1917-21; 1932; 1941-45	1914-18; 1940-45
Germany	1,187	6.95	942	242	3	1912-13; 1927-29; 1938-44	1915-17; 1919-20; 1923-24; 1931-34; 1946	1914-18; 1939-45
Italy	1,838	10.77	1,103	427	308	1909; 1915-18; 1929; 1937-42	1902; 1904; 1920-24; 1931; 1934; 1944-46	1915-18; 1940-45
Netherlands	1,397	8.18	1,081	179	137	1912-13; 1926-30; 1936-40	1908; 1916-1920; 1934; 1942-46	1940-45
Spain	2,178	12.76	1,272	337	569	1901; 1927-35; 1943-44	1905; 1910; 1917-21; 1936-39; 1941	1936-39
Sweden	2,139	12.53	1,739	371	29	1899; 1907; 1913-16; 1929-30; 1936-39	1905; 1918-19; 1921-22; 1932-33; 1941-45	None
Switzerland	1,680	9.84	504	310	866	1899; 1906; 1910-12; 1925-30; 1946	1903; 1917-22; 1936; 1941-44	None
Total	17,070	100.00	10,511	2,530	4,029			

Data source: SHARE waves 1, 2, and 4.

### Measures of Cognitive Functioning in SHARE

SHARE provides information on major domains of cognitive functioning, namely orientation, memory, executive function and language. We examine five indicators related to these domains. We dichotomize each single indicator and assign the lowest thirty percent of the distribution to the category “poor cognitive functioning” with the exception of the indicator “orientation in time”. Due to the left skewed distribution of this indicator the category of poor cognitive function consists of the lowest twenty percent (Table 2). We perform sensitivity analyses with different cut-points under the premise of covering similar and comparable sized groups.

**Table 2:** Descriptive statistics of the sample – basic model variables (N = 17,070).

Variable	Category	Number	Percent
Summary score	Poor	9,870	57.82
	Good	7,200	42.18
Orientation in time	Poor	3,351	19.63
	Good	13,719	80.37
Numeracy	Poor	4,961	29.06
	Good	12,109	70.94
Verbal fluency	Poor	5,179	30.34
	Good	11,891	69.66
Recall (1^st^)	Poor	5,287	30.97
	Good	11,783	69.03
Recall (2^nd^)	Poor	4,772	27.96
	Good	12,298	72.04
Gender	Male	7,830	45.87
	Female	9,240	54.13
Age	60–64	1,162	6.81
	65–69	3,665	21.47
	70–74	4,358	25.53
	75–79	3,942	23.09
	80–84	2,536	14.86
	85–89	1,035	6.06
	90+	372	2.18
Business cycle in year of birth (t)	Recession t	3,541	20.74
	Average t	7,169	42.00
	Boom t	6,360	37.26
Business cycle in year before birth (t−1)	Recession t−1	4,059	23.78
	Average t−1	7,159	41.94
	Boom t−1	5,852	34.28
Business cycle in year after birth (t+1)	Recession t+1	3,490	20.45
	Average t+1	6,853	40.15
	Boom t+1	6,727	39.41

Data source: SHARE waves 1, 2, and 4.

Orientation in time is measured by four questions about current day of the month, month, year, and day of the week. Every correct answer leads to one point, with a maximum of four points. We dichotomize the indicator distinguishing those with three or less correct answers from those who did not give any incorrect answer.

Recall ability is measured by a list of ten items where the respondent is asked which ones he or she remembers within one minute. The number of correct recalls is counted. We use quintiles when using the variable for the summary score. A maximum of four points are given when at least five items are recalled, followed by three points for four items, two points for three items, one point for two items, and zero points otherwise. Delayed recall ability is measured after the numeracy and verbal fluency tests. At that point, respondents are asked to repeat the recall. We dichotomize both items for their further analysis. First recall is differentiated into good (four to ten words) and poor recall ability (zero to three words), delayed recall into zero to one recalled words (poor) and two to ten recalled words (good). For the summary score, four points are given for at least four recalled items, three points for three items, and so on. [55] argue that the recall indicators are homogeneous across countries and cultures and hence enable analyses with cross-country data.

Numeracy ability is based on four questions that require simple calculations. The construction of the numeracy score is based on [2]. We dichotomize the indicator distinguishing those who cannot calculate ten percent of a number from those who are able to perform more complex calculations. Verbal fluency is measured by the respondent naming as many different animals as he/she can think of within one minute. For the single item analysis we dichotomized verbal fluency distinguishing those with zero to 13 words from those with 14 or more recalled words. For the construction of the summary score values are assigned according to quintiles: zero points are assigned if less than 12 animals are named, one point for 12 to 15, two points for 16 to 18, three points for 19 to 23, and four points for 24 and more animals.

We construct a summary score of cognitive functioning that ranges between 0 and 20 and consists of the sum of the points assigned in the individual indicators. The summary score is divided into the two categories above (15–20 points) and below the median (0–14 points). Our summary score follows the construction of the DemTect scale [56], a cognitive screening test of mild cognitive impairment and early dementia.

The three indicators verbal fluency, first, and second recall originate from the DemTect scale, while the indicator orientation in time stems from the Mini Mental State Exam, which is designed for the detection of Alzheimer dementia [57]. The indicator numeracy is widely used in economics and is described in [2].

The DemTect scale has a range of 0 to 18 points. A performance of 13 to 18 points is considered as adequate while 9 to 12 indicates mild cognitive impairment and 8 points or below indicates dementia. This means that in the DemTect scale the range of poor performance includes two-thirds of all possible points. With 0 to 14 of 20 possible points this is also true for our summary score. For the DemTect scale, a high validity of construction, and a high test-retest as well as inter-rater reliability was shown [56].

**Figure 1:**
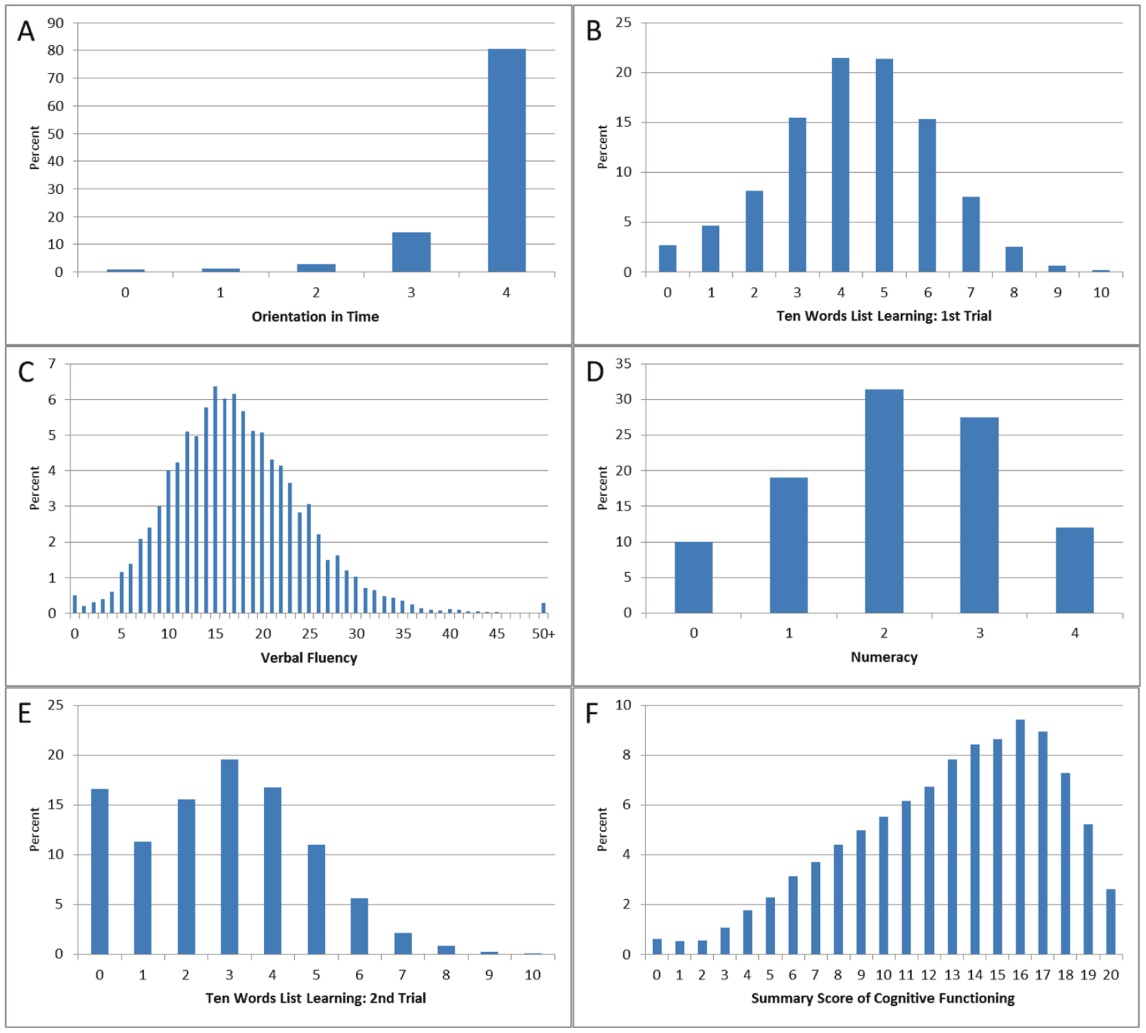
Distribution of the single items orientation in time (A), first recall (B), verbal fluency (C), numeracy (D), delayed recall (E), and summary score of cognitive functioning (F).

We perform sensitivity analyses using different cut-points for the individual indicators as well as for the summary score, but the results turn out to be insensitive. Figure 1 shows the percentage distributions of the single items orientation in time (A), first recall (B), verbal fluency (C), numeracy (D), delayed recall (E), summary score (F). The single items are all significantly correlated (SC-Spearman correlation, p = 0.00). The correlation is highest between immediate and delayed recall (SC = 0.72), followed by verbal fluency and the recall items (SC first recall = 0.53; SC delayed recall = 0.49). Numeracy is closely related to verbal fluency and the recall items (ranging between 0.42 and 0.47), while orientation in time is the least correlated with the other items.

### Economic Conditions at the Time of Birth

**Figure 2:**
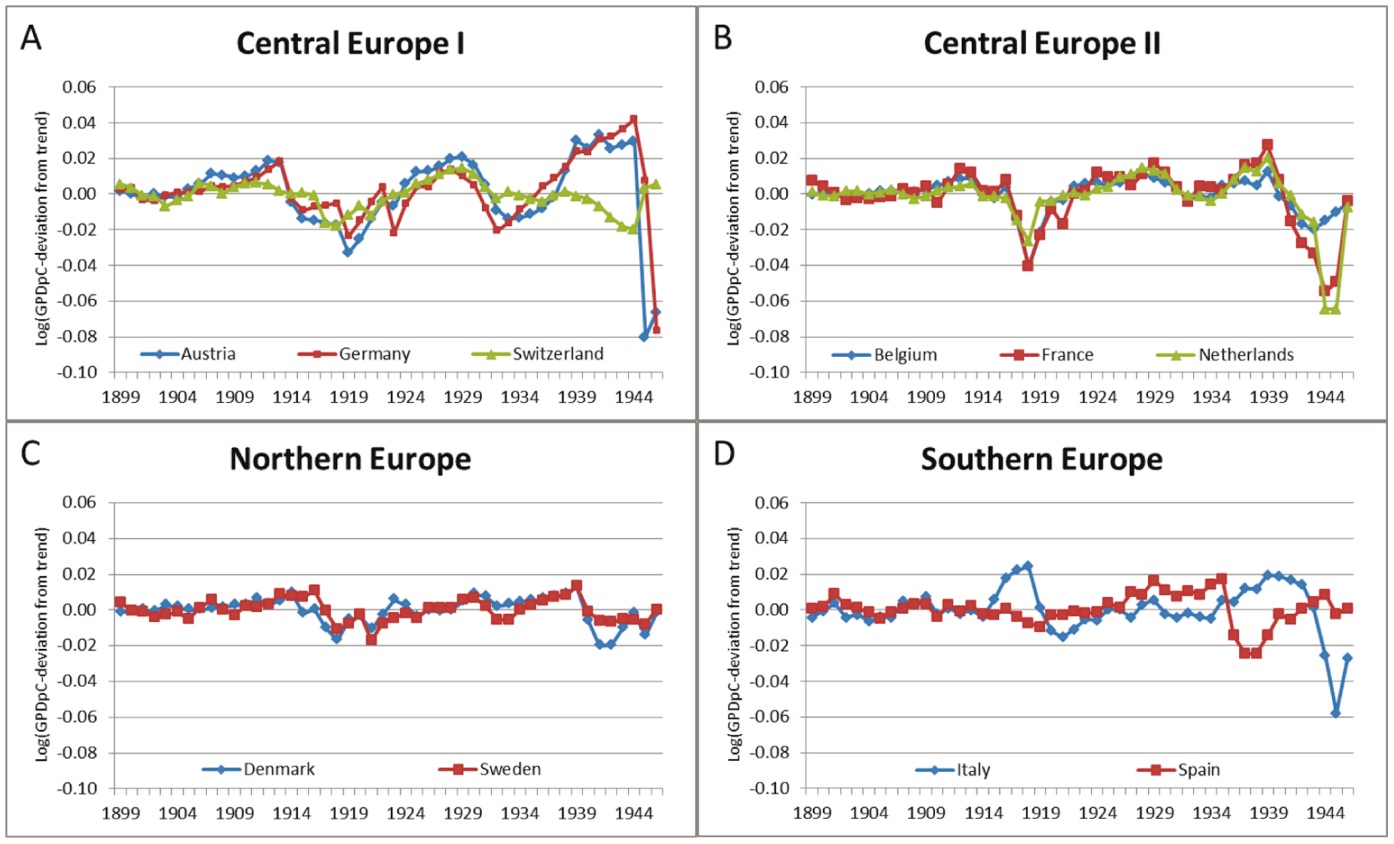
Cyclical components of log real GDP per capita for the ten SHARE countries.

Real GDP per capita is a widely used measure of aggregate economic conditions [8]–[10]. To capture idiosyncratic shocks in economic conditions we use the cyclical component of the natural logarithm of real GDP per capita at the country-level, applying the Hodrick-Prescott Filter [58] with a smoothing value of 500. The GDP data are based on [59]. Figure 2 shows the cyclical component of GDP per capita for the ten countries. Each cyclical component is transformed into one indicator with three categories. The category “recession” applies to those years that belong to the lowest quartile ( = 1^st^) of the country-specific cycle. The category “average” applies to the second and third quartile. The third category, “boom”, indicates years in the highest quartile ( = 4^th^). We link the year of birth to the cyclical component of that year *(t)*; see Table 1. We also run models where we include indicators for the years *t−1*, *t+1*, *t+3*, *t+10*, and *t+20*. Depending on the exact month of birth in year *t*, year *t−1* covers fetal development in-utero and the time before conception: for those born at the beginning of year *t*, it includes the time in-utero plus a maximum of three months before conception; for those born at the end of year *t*, it covers between 12 and 15 months prior to conception. Year *t+1* covers most of the first year of life for those born at the end of year *t*, and the second year of life for those born at the beginning of year *t*. The year *t+3* refers to early child development during toddler and pre-school age, the years *t+10* and *t+20* to early schooling age and working life at young adulthood.

The average age of the respondents born during recession periods is 74.16 years, whereas of those born during boom periods it is 73.63 years. Clearly, it is essential that our empirical analyses control for age. Moreover, we may omit or add certain birth cohorts to examine the sensitivity of the results. In particular, including war cohorts in the analyses attenuates the difference in mean age.

It is conceivable that less frail individuals are over-represented in birth cohorts born under adverse conditions. Such selectivity would bias our results towards zero (i.e., we would under-estimate a positive effect of favorable conditions at birth on cognitive ability later in life). It is known that dramatic shocks around birth, such as famines and epidemics, give rise to a fertility reduction especially among lower social classes. For instance, [60] showed that during the Dutch hunger winter 1944–1945 the fertility reduction was lower among groups of higher socioeconomic status. However, previous studies have found no systematic dependence of the size and the parental social-class composition of birth cohorts on the business cycle in European countries in the pre-1945 years. [61] examine this for the Netherlands, [62] for Sweden, and [10] for Denmark. In the Netherlands there was a slight reduction of the fraction of newborns among the highest social class in recessions, but leaving out that class does not affect the estimated long-run effect on late-life mortality. Notice that boom and recession periods in our time frame are very short (on average about 2 years), making it difficult for individuals to fine-tune their fertility behavior towards this. In addition, fertility control was less common or at least less effective than nowadays. To further investigate these issues, we examine the association between fertility and business cycle in our own data covering multiple countries and decades, and discuss the findings below.

In terms of income loss, modern recessions may not be as intrusive as in the pre-1945 years. However, it is not clear to what extent this applies to stress. On the one hand, many individuals may fear job loss during recessions. On the other hand, couples' working hours may be very high in boom years. In any case, notice that we use pre-1945 cycles as sources of exogenous variation to identify effects of which the existence does not depend on whether the particular sources we use still abound.

### Empirical Strategy

We use fixed effects regression models, to explore the effect of the business cycle on cognitive functioning for all countries combined. Since our data is clustered by country we use a robust cluster estimate of the variance. We specify a logit link function for the single items and the summary score and estimate equations of the form:
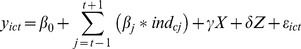
where *y_ict_* is a measure of cognitive functioning at age 60+ for individual *i* in country *c* born in year *t*, *ind_cj_* is the indicator for a recession, average or boom period in the country *c* and the years *j = t, t−1, t+1* as well as *t+3, t+10, t+20*. **X** is a matrix of individual level characteristics, **Z** a matrix of the country-level dummies, *β_0_, β_j_, γ*, and *δ* are the respective parameters and *ε_ict_* is the error term. We apply a nested modelling strategy. A set of first models includes as explanatory variables the indicator for the recession, average and boom periods in year *t*, age of the individual in five year age groups up to age 90+, sex as well as the country indicator. Having first-time respondents from the first, second as well as the fourth wave of SHARE means that we observe individuals from the same country with the same age who were in different stages of the business cycle at birth. This contrasts to a simple cross-sectional sample of individuals from a given country. With the latter type of sample, age effects are not identified from calendar time trends due to secular improvements in society, and a comparison between births from favorable and adverse years may be determined by age differences.

A second set of specifications includes the business cycle indicator for the year before and after the birth year, as well as for years *t+3*, *t+10*, *t+20* (not shown). Finally, a third set of specifications includes a set of covariates covering current socio-economic, demographic and health aspects of the individuals. We use education based on the International Standard Classification of Education (ISCED) as an indicator of socioeconomic position. Respondents with at least post-secondary education are assigned to the category high education whereas those with secondary education or less are assigned to low education. A third category comprises “refusal”, “don't know”, “still in school”, and “other”. Demographic information consists of partnership status and number of children. Health behavior is captured by body mass index (BMI) and smoking behavior. Disability is measured in terms of limitations in the activities of daily living (ADL) differentiating between respondents with at least one limitation and those with none. We use the EURO-D scale to measure depression which ranges from 0 (not depressed) to 12 (very depressed). We attribute depression symptoms to respondents with values four and above. Morbidity is represented by 14 binary indicators of chronic diseases, capturing whether a doctor ever told the respondent that (s)he has a certain disease. [63] assess the accuracy of such self-reported information in a Dutch sample of elderly individuals that is comparable to our data. They compare the information to data from general practitioners and conclude that the former information is fairly accurate. Interestingly, the level of accuracy is not influenced by the cognitive ability of the elderly respondents. Over-reporting of ill health as a justification for not working is common among labor force participants without work [64] but this does not seem to be an issue for those aged 60+. Table 2 and 3 give an overview of the distribution of the covariates.

**Table 3:** Descriptive statistics of the sample – complete model variables (N = 17,070).

Variable	Category	Number	Percent
Education	Low	14,233	83.38
	High	2,709	15.87
	Other/unknown	128	0.75
Family status	Spouse/partner	11,436	66.99
	Single	5,634	33.01
Number of children	0	1,637	9.59
	1	2,190	12.83
	2	3,972	23.27
	3	2,380	13.94
	4+	2,126	12.45
	No information	4,765	27.91
Body-mass-index	<18.5−underweight	261	1.53
	18.5–24.9−normal	6,421	37.62
	25–29.9 − overweight	7,188	42.11
	30 and above − obese	2,682	15.71
	Missing	518	3.03
Activities of daily living	No ADL limitations	14,541	85.18
	1+ ADL limitations	2,529	14.82
Depression symptoms	No	12,322	72.19
	Yes	4,588	26.88
	No information	160	0.94
Smoking	Yes, currently	1,851	10.84
	Never smoked	9,996	58.56
	Stopped	5,141	30.12
	Missing	82	0.48
Doctor told you had	Heart attack	3,105	18.19
Doctor told you had	Hypertension	6,718	39.36
Doctor told you had	High blood cholesterol	3,916	22.94
Doctor told you had	Stroke	912	5.34
Doctor told you had	Diabetes	2,177	12.75
Doctor told you had	Chronic lung disease	1,230	7.21
Doctor told you had	Asthma	728	4.26
Doctor told you had	Arthritis	4,509	26.41
Doctor told you had	Osteoporosis	1,387	8.13
Doctor told you had	Cancer	1,341	7.86
Doctor told you had	Stomach/duod./peptic ulcer	1,001	5.86
Doctor told you had	Parkinson's disease	181	1.06
Doctor told you had	Cataracts	2,611	15.30
Doctor told you had	Hip/femoral fracture	616	3.61

Data source: SHARE waves 1, 2, and 4.

The analyses require individuals to be alive at the time of their interview. Although the birth years are on average more recent than those used in typical long-run studies of early-life conditions (the vast majority of respondents being below age 75 at the time of the interview), it is of course a fact that a certain fraction of any birth cohort has died before the interview. This attrition plausibly leads to an overrepresentation of less frail (and more able) individuals within cohorts born under adverse conditions, which may bias our results towards zero [10].

## Results

Table 4 presents our main results in the form of odds ratios of good cognitive performance. Rows correspond to the separate analyses of the six dependent variables, and the two columns depict the effects of an average and boom period relative to a recession period in the year of birth *(t)*. For all indicators values above one indicate a higher likelihood of good cognitive functioning. The models control for the confounding effects of age, sex, and country. Below, when we refer to “boom periods” and “recession periods”, we tacitly omit the qualification that these are periods early in life rather than periods later in life. Clearly, we expect differences between those born in boom and recession years to be more pronounced than differences between either of these two groups on the one hand and those born in average years on the other. However, the latter group is larger in number, and in some cases, when the contrast boom vs. recession does not give rise to a significant effect, the contrast boom vs. (recession + average), and/or the contrast (boom + average) vs. recession, gives rise to effects that are significantly different from zero.

**Table 4:** Odds ratios of good cognitive functioning of an average or boom birth year (year t); recession is the reference category.

Dependent variable	Business cycle in the year of birth (t)	
	Average	Boom
	**BASIC MODELS**	
Summary score	1.109**	1.237***
	(0.050)	(0.084)
Orientation in time	1.022	1.046
	(0.070)	(0.047)
Numeracy	1.038	1.192**
	(0.038)	(0.088)
Verbal fluency	1.000	1.066
	(0.052)	(0.087)
Recall (1st)	1.052	1.136*
	(0.064)	(0.082)
Recall (2nd)	1.027	1.110
	(0.066)	(0.075)
	**COMPLETE MODEL**	
Summary score	1.122***	1.250***
	(0.048)	(0.079)

Data source: SHARE waves 1, 2, and 4; all cells contain odds ratios reported from logit regression models. Robust standard errors are in parentheses. Basic models control for sex, age and country. The complete model in addition controls for education, family status, number of children, BMI, ADL, depression, smoking and chronic diseases. ***p< = 0.01; **p< = 0.05; *p< = 0.1.

The estimated effects of boom, average and recession periods in the year of birth *(t)* follow our expectations insofar that booms implicate higher chances of good cognitive functioning late in life than recessions.

Being born in boom years increases the likelihood of good cognitive functioning in terms of numeracy by an odds ratio of 1.19 (p = 0.02). Of verbal fluency the odds ratio is 1.07 (p = 0.43), of the first recall it is 1.14 (p = 0.08) and of the second recall 1.11 (p = 0.12). Results for average periods are intermediate. Combining all indicators into the over-all summary or omnibus score, the odds ratio is 1.24 (p = 0.00) for boom and 1.11 (p = 0.02) for average periods.

Our second set of model specifications includes cyclical indicators for the year prior to birth *(t−1)* and the year after birth *(t+1)* in addition to the year of birth *(t)*. The results above for the year of birth *(t)* remain stable, though the effect on numeracy loses statistical significance (Table 5). Turning to the year prior to birth booms show no significant effects on the indicators except delayed recall (OR = 1.14; p = 0.01). The year after the birth year also shows no effect of the boom periods.

**Table 5:** Odds ratios of good cognitive functioning of an average or boom birth year (year t), year before birth (year t−1), and year after birth (year t+1); recession is the reference category.

Dependent variable	Business cycle					
	year of birth (t)		year before birth (t−1)		year after birth (t+1)	
	Average	Boom	Average	Boom	Average	Boom
	**BASIC MODELS**					
Summary score	1.108	1.208**	0.972	1.039	0.990	0.986
	(0.069)	(0.096)	(0.067)	(0.069)	(0.035)	(0.061)
Orientation in time	1.018	1.010	0.890*	0.978	1.083	1.091
	(0.093)	(0.075)	(0.050)	(0.061)	(0.078)	(0.095)
Numeracy	1.108	1.130	1.030	1.096	1.021	1.000
	(0.035)	(0.095)	(0.080)	(0.082)	(0.030)	(0.061)
Verbal fluency	1.035	1.142	0.960	1.039	0.960	0.914
	(0.071)	(0.136)	(0.057)	(0.100)	(0.086)	(0.061)
Recall (1^st^)	1.070	1.185**	0.999	1.021	1.007	0.919
	(0.065)	(0.101)	(0.068)	(0.100)	(0.042)	(0.071)
Recall (2^nd^)	1.002	1.065	1.041	1.115**	1.071	0.989
	(0.082)	(0.094)	(0.052)	(0.060)	(0.050)	(0.053)
	**COMPLETE MODEL**					
Summary score	1.127*	1.233**	0.963	1.018	0.982	0.988
	(0.072)	(0.105)	(0.066)	(0.065)	(0.036)	(0.062)

Data source: SHARE waves 1, 2, and 4; all cells contain odds ratios reported from logit regression models. Robust standard errors are in parentheses. Basic models control for sex, age and country. The complete model in addition controls for education, family status, number of children, BMI, ADL, depression, smoking and chronic diseases. ***p< = 0.01; **p< = 0.05; *p< = 0.1.

When we run separate models (not shown) for the years *(t−1)* and *(t+1)* we find positive effects for boom periods in the year prior to birth *(t−1)* that are similar to those in the year at birth *(t)*. No significant effects exist for the year after birth *(t+1)*.

We do not find any consistent and significant business cycle effects for early child and toddler years *(t+3)*, early school years at time *t+10*, and early adulthood at time *t+20* (results not shown).

Effect sizes and significance of the business cycle indicator remain stable when current social, demographic and familial characteristics are introduced in the models. This is also true for the risk factors and the health characteristics of the respondents, which have been selected according to earlier studies about cognitive functioning and dementia [54]. For the over-all omnibus score this is shown in Table 6 (“Complete Model”). We also estimate the basic logit model for the over-all summary score for each country separately (results not shown). In the light of the small sample size per country, it is not surprising that the estimates of interest are not always significantly different from zero. Only for Switzerland and Austria does the estimated effect of birth in a boom year (as compared to birth in a recession year) not have a value above one. In terms of odds ratios, the strongest effects are for Germany (1.98, p = 0.03), Italy (1.93, p = 0.03), Sweden (1.40, p = 0.06) and Belgium (1.33, p = 0.14). However, differences between countries are not significant at the conventional significance levels.

**Table 6:** Odds ratios of good cognitive functioning based on the summary score of cognitive functioning.

Variable	Category	Odds ratios	Robust SE
Gender (RG: Males)	Females	1.130*	0.079
Age (RG: 60–64)	65–69	0.535***	0.054
	70–74	0.396***	0.028
	75–79	0.243***	0.019
	80–84	0.163***	0.013
	85–89	0.094***	0.011
	90+	0.063***	0.015
Business cycle in the year of birth (t) (RG: Recession_t_)	Average_t_	1.122***	0.048
	Boom_t_	1.250***	0.079
Education (RG: Low)	High	3.204***	0.364
	Refusal/don't know/still in school/other	1.380	0.407
Family status (RG: Spouse/partner)	Single	0.826***	0.039
Number of children (RG: 0)	1	1.050	0.076
	2	1.191***	0.056
	3	1.143***	0.055
	4+	0.937	0.082
	No information	1.086	0.096
BMI (RG: <18.5−underweight)	18.5–24.9−normal	1.366	0.299
	25–29.9− overweight	1.332	0.311
	30 and above − obese	1.269	0.323
	Missing	0.688	0.162
ADL (RG: No ADL limitations)	1+ ADL limitations	0.555***	0.044
Depression symptoms (RG: No)	Yes	0.536***	0.031
Smoking (RG: Yes, currently)	Never	1.013	0.057
	Stopped	1.186***	0.063
	No information	0.923	0.293
Doctor told you had (RG: No)	Heart attack	0.912	0.059
Doctor told you had (RG: No)	Hypertension	0.986	0.044
Doctor told you had (RG: No)	High blood cholesterol	1.067**	0.032
Doctor told you had (RG: No)	Stroke	0.601***	0.058
Doctor told you had (RG: No)	Diabetes	0.798***	0.056
Doctor told you had (RG: No)	Chronic lung disease	0.918*	0.044
Doctor told you had (RG: No)	Asthma	0.886	0.110
Doctor told you had (RG: No)	Arthritis	1.102	0.094
Doctor told you had (RG: No)	Osteoporosis	0.998	0.101
Doctor told you had (RG: No)	Cancer	1.162***	0.062
Doctor told you had (RG: No)	Stomach/duodenal/peptic ulcer	1.119	0.120
Doctor told you had (RG: No)	Parkinson's disease	0.518***	0.129
Doctor told you had (RG: No)	Cataracts	1.142***	0.052
Doctor told you had (RG: No)	Hip/femoral fracture	1.016	0.051

Data source: SHARE waves 1, 2, and 4; controlled for sex, age and country. Robust SE: robust standard errors. ***p< = 0.01; **p< = 0.05; *p< = 0.1.

If we include war cohorts, resulting in a lower mean age of those born in recessions, then the advantageous effects of birth in booms are somewhat smaller in magnitude but they are still significant. Thus, the results are not driven by individuals born in booms having benefited more from secular improvements in society than individuals born in recessions.

## Discussion

The existence of an economic boom during the year of birth increases the risk of good cognitive functioning at age 60 and above while recessions tend to impair late-life cognitive functioning. In our study, all four domains of cognitive functioning, represented by five indicators, and the summary score, follow this pattern. Adding simultaneously economic information for the year prior to birth and the year after birth, as well as three, ten and twenty years after birth does not yield significant results and changes the effect of the economic situation in the year of birth only marginally.

While the mechanisms underlying the effect of boom and recession periods on late-life cognition cannot be easily determined, a series of possible links exist that are closely related to the present knowledge about causal pathways from early-life conditions to late-life health outcomes. Boom and recession periods plausibly differ in terms of the quality and quantity of nutrition as well as the psychological stress level in the household. In addition, differences in the extent of crowded housing and access to health care might create differences in disease exposure. Nutrition, disease exposure and stress early in life have all been connected to health outcomes late in life, including mental outcomes (recall the discussion of the background literature).

The economic effect on pre-natal and early natal nutrition is likely to be of major importance for cognitive functioning [27]. Recessions before 1945 involved income loss for many households. As discussed earlier, biological cues transmitted early in life may permanently modify the metabolic development, affecting cognitive abilities later in life. The cardiovascular and obesity effects of reduced nutrition in utero have been shown to be stronger if the affected individuals are exposed to a more favorable environment later in childhood [65]. The latter by construction applies to the business cycle, since any recession is sooner or later followed by a boom.

In addition to direct nutritional effects, it is likely that economic hardships, the fear of hardships and the loss of employment and income in the near future increase the level of psychological stress in the household. Exposure to this in utero or shortly after birth may be neurodegenerative in such a way that cognitive abilities decline at high ages [66]. Alternatively, more adverse socioeconomic conditions [67] and a harsher family climate in early childhood [68] may result in a heightened risk of chronic (cardiovascular) health problems and cognitive limitations later in life [68]–[71].

The medical history characteristics of the respondents yielded the expected results. The presence of stroke or diabetes significantly reduces the likelihood of good cognitive functioning. No effects exist for hypertension and body-mass-index while hypercholesteremia seems to have favorable effects. When interpreting these results one has to keep in mind that diabetes, obesity, high blood pressure and hypercholesteremia are highly correlated, constituting the so-called metabolic syndrome [72]. Net effects are therefore difficult to quantify. In addition, dementia is associated with weight loss [73] which positively affects other diseases such as the metabolic syndrome. The presence of ADLs and depression significantly reduces the risk of good cognitive functioning by about a half. This is also true for Parkinson's disease. Interestingly, there is evidence that cancer significantly increases the risk of good cognitive functioning, which is supported by earlier findings, that cancer survivors have a lower risk of developing dementia [74]. Those who stopped smoking have better cognitive functioning which, again, supports findings that current smoking increases the risk of poor cognitive functioning [75]. It is interesting to note, that the effect size of the business cycle is slightly larger than that of stopping smoking.

The existence of social networks, here measured in terms of living with a spouse or partner and the number of children, positively influences cognitive functioning. The U-shaped effect for children has already been reported in earlier studies on mental health, with no child and more than four children reducing mental health at old age [48]. Overall, the results of the medical history and social network characteristic, while interesting on their own, support the validity of our summary score.

As explained earlier, our methodology requires that the composition of newborns does not vary systematically over the business cycle. We may investigate this by examining the association between fluctuations in fertility and the business cycle, following the idea that such an association is indicative of systematic changes in the underlying composition. We carefully examine the correlation between the number of births and the business cycles using birth data from the human mortality database (www.mortality.org) for the period 1900 to 1945 excluding war years. No data are available for Germany. We do not find any significant relationship once we control for secular trends. This reinforces our claim that in our study period, economic cycles do not lead to selective fertility to the same extent as famines and epidemics may do. Since we exclude war periods, the Dutch hunger winter is not part of this study.

One limitation of this study is that GDP is only measured on an annual basis. We may loosely interpret the cyclical effects at the birth year and surrounding years as effects at various developmental stages: the effect at *t−1* covering influences prior to conception and in utero, the effect at *t* combining developmental stages ranging from pre-conception to the first year of life, and the effect at *t+1* covering parts of the first and second years of life. The overlapping nature of the three periods explains why some effects at *t* become insignificant once we control for conditions at *t−1* and *t+1*. Assigning the value of the business cycle of the previous year to all those born between January and the end of June does not change the overall result. The implication of the lack of preciseness in the timing of the cyclical indicator in relation to the date of birth is that we cannot identify critical periods. Our results suggest that the whole period, ranging from pre-conception to the first year of life, is important for late-life cognitive functioning. Testing the effect of the business cycle on other periods of life such as three, ten, and twenty years after birth does not yield significant results.

The indicators of cognitive functioning we use in our study may be influenced by schooling access and job opportunities in adulthood. Studies suggest that being in a cognitively-demanding job is both predicted by prior cognitive ability, but may also protect from cognitive decline. [76] provide a comprehensive review of studies exploring this pathway; however, in their own study they do not find evidence for a protective effect of cognitively-demanding jobs. One might argue that one cannot necessarily attribute all the effects of the economic cycle to circumstances at birth but that social pathways may play an important role. We cannot test these pathways with our data since we do not have information on prior cognitive abilities. Our complete model, however, includes education which is an important determinant of job-opportunities and protects from cognitive decline. We find that education itself exerts an important effect on cognitive functioning late in life but does not explain or modify the effect of the business cycle. This supports our conclusion that the economic situation at the time of birth has long-run implications for late-life cognition that cannot be simply explained by pathways through schooling access and job opportunities. Our finding that the business cycles at times *t+3*, *t+10*, and *t+20* do not have long run effects on cognition further strengthens this conclusion.

Another limitation is that as yet no single causal mechanism from economic conditions early in life to health later in life has been identified. Given the possibility of various pathways, however, this can also be seen as an advantage of the economic indicator. It highlights the importance of health, family and social policies directed towards women who want to become mothers, as well as towards pregnant mothers and young children. In times of economic hardship these groups need special support to avoid negative long-term consequences on the cognitive abilities of the new generation.
